# Supplementary material for: Skilled birth attendance and its associated factors in Chad and Nigeria: A multilevel analysis of DHS data
Source: PLOS Glob Public Health. 2025 Dec 10;5(12):e0005290. doi: 10.1371/journal.pgph.0005290 (PMC12694868; doi:10.1371/journal.pgph.0005290)
Supplement: Attachment — Submitted filename: Point by Point response.docx [file pgph.0005290.s005.docx]

**Date: September, 2025**

To: **PLOS Global Health**

**Authors’ Point-by-point response to editor and reviewer comments**

We are very grateful to both the editor and reviewers for your comments and suggestions. All the concerns raised so far will have an undeniable impact on improving the quality and readability of our scholarly work. Appreciating your effort and valuable comments, we have provided possible reflections and amended the raised concerns and questions. Kindly find our reflections here. We hope you will consider the revised manuscript acceptable for publication in **PLOS Global Health,**

|  | **Reviewers Comment** | **Authors Response** |
| --- | --- | --- |
|  | Reviewer 1 | Response |
| 1. | Strength • It shows the urgency of global and national level intervention to improve SBA and MMR in extremely high maternal mortality countries • It indicate the statistical methods used to analyze DHS data due to it hierarchical nature | We thank the reviewer for highlighting these strengths. The study underscores the urgency of interventions to improve SBA and reduce maternal mortality in high-burden countries and demonstrates robust multilevel analysis accounting for the hierarchical DHS data structure. |
| 2. | Weakness • The word “extremely high maternal mortality countries” make the title vague in terms of geographic coverage; better if the two counties are mentioned (Chad and Nigeria) • the orders in the title needs improvement; for decision or policy makers it is better if the outcome comes first rather than statistical methods “Outcome → Determinants → Context → Method • Multilevel analysis of what type of data? cross sectional study or DHS data better if it indicated in the title “Multilevel analysis of DHS data” Overall Recommendation and title suggestion Better if the authors rephrase it again “Skilled birth attendance and its associated factors in Chad and Nigeria: A multilevel analysis of DHS data” | We thank the reviewer and have revised the title for clarity and policy relevance as: “Skilled birth attendance and its associated factors in Chad and Nigeria: A multilevel analysis of DHS data.” **Page 1 line 1-2.** |
| 3. | Trends in maternal mortality estimates 2000 to 2023 report; Estimates by WHO, UNICEF, UNFPA, World Bank Group and UNDESA / Population Division The report said that in 2023, no countries were estimated to have had “extremely high” maternal mortality; the data for the Nigeria and Chad are 993 and Chad 748 MMR respectively. Your reference number 17 about the UNICEF data about maternal mortality also showed this number | We thank the reviewer for their comment. The manuscript now clarifies that the previously cited estimates (Nigeria 1,047; Chad 1,063) refer to WHO data from 2017–2020, used here to illustrate recent historical trends. We have also added the 2023 WHO/UNICEF/UNFPA/World Bank/UN DESA estimates (Nigeria 993; Chad 748) to reflect the most current maternal mortality data. Kindly see the revised manuscript on **page 4 line 46-49.** |
| 4. | Abstract Strength • Objectives, Methods, Results, and Conclusions clearly included • Objective: it is well stated • Methods: use of DHS data and statistical methods well mentioned (CI and P-value)  • Results: Key findings are well presented with adjusted odds ratios and confidence intervals.  • Conclusion: actionable recommendations are included for respective bodies | We thank the reviewer for the positive feedback. We are glad that the objectives, methods, results, and conclusions are clear, and that the use of DHS data, statistical methods, key findings with AORs and CIs, and actionable recommendations are appropriately presented. |
| 5. | Weakness • Lines 16-17:- the data in the introduction lacks a specific reference year; please indicate the year or time frame for this global estimate. The data of the two countries or actual MMR are less than 1000 in 2023 report; better if precise figures are used to avoid potential misinterpretation. Globally, an estimated 260 000 women died from a maternal cause in 2023. | We thank the reviewer for the comment. We have **updated the introduction to include the reference year and precise figures**, stating that in 2023, **Nigeria and Chad had maternal mortality rates of 993 and 748 per 100,000 live births, respectively**, and that **globally an estimated 260,000 women died from maternal causes**. This ensures accuracy and prevents potential misinterpretation. Page 4 line 47-49. |
| 6. | • “The sentence from line 19-21 is redundant. It is already addressed in the aim. deleted | Thank you for the suggestions. We have deleted the redundant text. Page and line N/A. |
| 7. | • The phrase “extremely high maternal mortality countries” is repeated three times in the background paragraph. Better if removed or rephrased (line 18, 20 & 23 | Thank you. We revised the repeated words. Kindly see the introduction section of the manuscript from page 4-6. |
| 8. | Line 24:- It does not clearly shows the survey year of the two countries. Please specify the years of DHS data The word “expected” is not strong for conclusion or recommendation for the government. It should be mandatory priority for national health systems | Thank you for the comment. We revised and included the survey year. Kindly see the revised manuscript on page 2 line 21-22. |
| 9. | Background Line 49:- Need reference year as indicated above. Providing the reference year for each data point will improve transparency.  Line 53-55:- the sentence globally, in 1990, the maternal mortality rate was 385 deaths per 100,000 live births, but in 2015, it was 216 deaths per 100,000 live births. I think this data is outdated and better to use update data from the latest WHO report data to improve the timeliness and accuracy of the introduction. (The global MMR in 2023 was estimated at 197 maternal deaths per 100 000 live births) | Thank you for the comment. We revised the outdated data. Kindly see the revised manuscript on page 4 line 46-49. |
| 10. | Line 73-75: The sentence “However, this goes in the opposite direction in Chad and Nigeria, where more than 1000 MMR per 100,000 live births still continued (17)”. I think the data of the two countries cited in reference 17 appears to be inconsistent. The data for the year 2023 indicate Nigeria 993 and Chad 748. The data from 2017 to 2020 which is Chad 1063 and Nigeria 1047 cited in reference (10, 11). If it for consistency with DHS survey year clarifying this will improve data transparency and ensure consistency between cited statistics and referenced sources | We revised it accordingly. Kindly see the revised manuscript on page 6 line 98-105.  |
| 11. | The term “skilled birth attendance” and its abbreviation “SBA” are used interchangeably across the manuscript. This inconsistency may confuse readers and disrupt the flow of the text better to make it uniform | Well revised and made uniform. Kindly see the revised manuscript. |
| 12. | Methods Line 134: Please verify the source and specify the reference year for the >1000 figure | Thank you. Here are the two sources to align with the DHS survey year.  1. World Health Organization. (2021). Maternal mortality in Africa: A head-cracking matter. WHO Regional Office for Africa. Retrieved from <https://aho.afro.who.int/blogs-detail/af?id=77>.  2. World Health Organization Regional Office for Africa. (2020). Maternal mortality regional factsheet. Retrieved from <https://files.aho.afro.who.int/afahobckpcontainer/production/files/iAHO_Maternal_Mortality_Regional_Factsheet.pdf>.  We clearly stated in the revised manuscript on page 6 line 98-105 |
| 13. | Line 134: The importance of Kids Record (KR) dataset to extract factors associated with SBA was mentioned. It is better if they mention its importance beyond identifying factors. | Thank you. Well explained in the revised manuscript on page 7 line 112-118. |
| 14. | Line 137: Please specify the years of DHS survey used for Chad and Nigeria in this study | This study used the most recent Demographic and Health Survey (DHS) datasets for Chad (2014–2015) and Nigeria (2018) to analyze factors associated with skilled birth attendance. Page 6 line 98-99. |
| 15. | **Study Variable** Media Exposure Line 150-151: Instead of repeating the above exposure sentence you can rephrase it as “those with no access to any were considered unexposed. | We accepted and revised page 7 line 130. |
| 16. | **Modeling approaches** Line 179: Better if you explain the importance of each model in the analysis of this study. Why we use the null model? Example the null model includes only random intercepts and used to assess baseline cluster level variance in SBA  Line 179: What is the role of CCI in this study beyond indicating the presence of random effects? | Thank you for your comment. We have revised the section to explain the purpose of each model in our multilevel analysis. *page 9 line154- 177* |
| 17. | Result Line 179: Table 1: *Media exposure what (*) these indicate? It is not explained under the table | Thank you for noting this. The asterisk (*) in Table 1 under “Media exposure” was an editing error and had no intended meaning. We have now removed it from the table to avoid confusion. Kindly see table 1. |
| 18. | Line 182: Better if the prevalence of individual counties mentioned for ease of understanding in the sentence rather than presenting pooled estimates | Thank you for this suggestion. We would like to note that the prevalence of skilled birth attendance for Chad and Nigeria is already presented separately in Figure 1. However, to improve clarity in the text, we have also revised the sentence to explicitly mention the country-specific prevalence alongside the pooled estimate. |
| 19. | Line 195: I think it is better to include “AOR” in this section since you are reporting for the first time in this part | Thank you for this helpful comment. We have revised the results section to specify that the reported estimates are **Adjusted Odds Ratios (AOR)** since this is the first time they are introduced in the text. Page 9 line 167. |
| 20. | Line 195-208: The phrase “reproductive-age women” is repeated eight times within a single paragraph—which disrupts the flow and readability of the text. I recommend if they use pronouns or simplified phrase | Thank you for pointing this out. We have revised the results section to avoid repetition of the phrase “reproductive-age women” by using pronouns (e.g., “they,” “these women”) and alternative expressions (e.g., “respondents,” “participants”). This improves the readability and flow of the text. Page 13 line 209-219. |
| 21. | Discussion Line 210: During the discussion of the finding based on DHS survey conducted at different time (Chad 2014–15, Nigeria 2018 if the years are correct) the observed differences in skilled birth attendance between Chad and Nigeria should be interpreted with caution due to the temporal gap in DHS survey implementation. Time variation between surveys can introduce confounding effects that influence health system performance indicators, including skilled birth attendance. | Thank you for this insightful comment. We have now revised the discussion to clarify that differences in skilled birth attendance between Chad and Nigeria should be interpreted with caution due to the temporal gap in DHS survey implementation (Chad 2014–15, Nigeria 2018). We also highlighted that changes over time may confound observed differences in health system performance indicators. Page 16 line 222-233 |
| 22. | Line 210: the phrase Sub Saharan Africa and the abbreviation SSA should be used consistently | We thank the reviewer for this important note. We have revised the manuscript to use “Sub-Saharan Africa (SSA)” consistently, introducing the abbreviation at first mention and using “SSA” thereafter for clarity. |
| 23. | Line 239: In most of academic writings “odds” is treated as a plural noun. I think better to consider the grammar across the manuscript. Example Odds of skilled birth attendance were..." | We appreciate the reviewer’s observation. We carefully reviewed the manuscript and corrected grammatical inconsistencies by treating “odds” as a plural noun. Kindly see the revised manuscript. |
| 24. | Line 239: Why different citation style is used in this sentence? Consistency in citation is mandatory throughout the entire document. In academic writing, using different citation styles within the same manuscript is generally not acceptable | We thank the reviewer for this careful observation. We have revised the manuscript to ensure consistency in citation style throughout. Specifically, we corrected the sentence at Line 239 where a different citation format was mistakenly used. Now, all references follow the uniform PLOS ONE numeric citation style. |
| 25. | Line 240: The discussion of odds ratio started with educational factors; from the lower it is at third stage. I think it is better if you start with the most influential factors first (e.g., starting with ANC visit: OR 5.56) this may help the policy makers to give priority for intervention based on the study findings Odds of Skilled Birth Attendance (Increasing Order) Factor Odds Ratio (OR) 95% Confidence Interval (CI) Middle wealth status 1.37 (1.23 – 1.52) Media exposure 1.5 (1.38 – 1.63) Primary education 1.77 (1.62 – 1.95) Rich wealth status 2.11 (1.87 – 2.38) High community education level 2.73 (2.26 – 3.29). Secondary education 4.06 (3.59 – 4.57). ANC attendance 5.56 (5.03 – 6.14) | Thank you for this insightful comment. We have revised the discussion to begin with antenatal care (ANC) attendance, which showed the highest odds of skilled birth attendance (OR = 5.56). Other predictors are now discussed in descending order of strength, from secondary education to middle wealth status. This reorganization highlights the most influential factors first, which may guide policymakers in prioritizing interventions to improve SBA. Kindly see line 257-310. |
| 26. | Line 333: Data Availability Statement: The data used in this study are publicly available from the Demographic and Health Surveys (DHS) Program through a formal request process through the DHS  website: <https://dhsprogram.com/data>. I think this should be clearly stated. My question; is the sentence appropriate for DHS survey data from ethical point of view? Example of the Trend of MMR in 2023 For the sentence “International Children’s Fund (UNICEF) reported that South Sudan, Chad, and Nigeria had extremely high maternal mortality rates (>1000) in 2023. The picture may be used as reference African countries with the highest maternal mortality rate in 2023(deaths per 100,000 live births) | Revised 364. |
| 27. | Overall, the manuscript is well written; however, if the above points are incorporated, it will further enhance its clarity | Thank you once again for your appreciations. |
| 28. | Reviewer 2 major |  |
| 29. | Why is the prevalence of skilled birth attendance almost 30% higher than previously reported? | Thank you for your comment. The observed prevalence of skilled birth attendance in our study is higher than some previously reported figures due to several reasons:   1. **Use of the most recent DHS data:** We utilized the latest available datasets (Chad 2014–15, Nigeria 2018), which may reflect improvements in SBA coverage since earlier reports. 2. **Inclusion/exclusion criteria:** Our study population included only women aged 15–49 with recent live births within the survey period, which may differ from prior studies using broader or different samples. 3. **Definition of SBA:** Differences in how skilled birth attendance is defined or categorized across studies can lead to variability in prevalence estimates. We strictly followed the DHS definition of SBA for consistency. 4. **Data weighting and survey design:** Our analysis accounted for the DHS sampling weights and multistage cluster design, which can slightly adjust prevalence estimates compared to unweighted or differently weighted analyses.   These factors together likely explain why our reported prevalence is approximately 30% higher than some earlier reports. |
| 30. | • There is a contradiction in that although the prevalence of SBA is higher in Nigeria (Fig. 1), table 3, model 3, suggests that the adjusted odds for SBA is higher in Chad than Nigeria. This needs to be discussed.  • There appears to be no discussion of the effects of community level variables. Then what’s the point of including these in your models?  Would any of the conclusions have changed if you had used only individual level variables in your model? | Thank you for pointing out this important observation. We acknowledge the apparent discrepancy: while the raw prevalence of skilled birth attendance (SBA) is higher in Nigeria (as shown in Figure 1), the adjusted odds in Model 3 are higher for Chad. This difference arises because the adjusted odds account for individual- and community-level factors, such as maternal education, wealth, urban/rural residence, and community characteristics.  In other words, after controlling for these covariates, the likelihood of SBA is relatively higher in Chad compared to Nigeria, suggesting that contextual and compositional differences between the countries affect the adjusted estimates. This highlights that raw prevalence alone may not reflect the underlying determinants of SBA, and multilevel analysis provides a more nuanced understanding of country-specific factors influencing skilled delivery. **Page 16 and Line 224-233.** |
| 31. | • Is SBA self reported by mothers? If yes, can there be variations in respondents understanding of what SBA means? If yes, how does this affect results? | Thank you for your question. Yes, skilled birth attendance (SBA) is self-reported by mothers in the DHS surveys. We acknowledge that there may be some variation in respondents’ understanding of what constitutes a skilled birth attendant. This potential variation introduces recall or reporting bias, which may slightly affect the accuracy of prevalence estimates.  However, the DHS standardizes questions and provides clear definitions in its survey instruments, which helps minimize misclassification. Additionally, any residual variation is expected to be non-differential, meaning it would likely bias results toward the null rather than create spurious associations. Therefore, while self-reporting may introduce minor variability, the overall patterns and associations observed in our analysis are likely robust. |
| 32. | Technical comments:  • How was the Kids Record linked to the survey data? Was there a unique identifier? | Sure. this is a unique identifier based on the DHS guideline. In the DHS datasets, the **Kids Record (KR) file is linked to the household and individual women’s data using unique identifiers**. Page 7 Line 108-118 |
| 33. | • How were the community level clusters created? Provide details on geographic location and clustering algorithm. | Described in the revised manuscript on page 7 line 108-110. |
| 34. | • Howe correlated are the predictors? Is multicollinearity a problem, i.e. are adjusted odds sensitive to the presence of correlated variables in the model? | Well described in the revised manuscript. Kindly see on page 8 line 154-158. |
| 35. | • What do the weights in modelling depend on? Provide more details. | Thank you for the comment. All analyses were weighted using the DHS individual sampling weights (v005), which adjust for unequal probabilities of selection and non-response, ensuring nationally representative estimates. The multilevel models incorporated these weights while also accounting for clustering at the community level (PSU) and stratification by region. Kindly see the revised manuscript on page 8 line 151-155 |
| 36. | • What is the bivariable analysis? Having model equations would be helpful for understanding. | Bivariable logistic regression was conducted for each independent predictor to examine its individual association with skilled birth attendance. Variables with p < 0.05 were considered for inclusion in the multivariable multilevel logistic regression. We explained the equation well in the revised manuscript. Kindly see the revised manuscript on page 8 line 159-165 |
| 37. | • Consider the use of AIC instead of deviance, as models have different numbers of parameters. | Thank you for your compliant. However, we chose deviance over AIC because our focus was on nested model comparisons rather than non-nested models with differing numbers of parameters. We compared nested multilevel logistic regression models using deviance statistics. The difference in deviance between models follows a chi-square distribution, allowing us to evaluate whether adding predictors significantly improved model fit. |
| 38. | Minor comments |  |
| 39. | • Keep table headers on same page as table contents. | Thank you for your suggestion. We have revised all tables so that table headers now remain on the same page as the table contents, improving readability and ensuring compliance with journal formatting guidelines. |
| 40. | • Table 3: where is the bivariable model? | Thank you for your comment. We considered including bivariable (unadjusted) odds ratios in Table 3; however, doing so would make the table too compact and reduce its visual clarity. To maintain readability and focus on the main findings, we have presented only the adjusted (multivariable) odds ratios. The bivariable analyses were conducted as part of model building and are described in the Methods section. |
| 41. | • Is Fig. 1 necessary? Can’t that data be incorporated in Table 2? | Thank you for your comment. We included Figure 1 to provide a visual overview of the prevalence of skilled birth attendance across countries and regions, which allows readers to quickly grasp patterns and differences. While the data could technically be included in Table 2, presenting it as a figure enhances readability and visual appeal, making it easier to interpret trends and regional variations briefly. Therefore, we believe Figure 1 is necessary for effective communication of the findings. Kindly see figure 1 in the revised manuscript. |

Thank you so much for all your comments.
