# Supplementary material for: Skilled birth attendance and its associated factors in Chad and Nigeria: A multilevel analysis of DHS data
Source: PLOS Glob Public Health. 2025 Dec 10;5(12):e0005290. doi: 10.1371/journal.pgph.0005290 (PMC12694868; doi:10.1371/journal.pgph.0005290)
Supplement: Attachment — Submitted filename: Revised Comments PGPH-D-25-02012R1_ Review Report_SBA.docx [file pgph.0005290.s004.docx]

# Reviewed Summary– Manuscript PGPH-D-25-02012R1

I have reviewed the revised manuscript titled *“Skilled birth attendance and its associated factors in Chad and Nigeria: A multilevel analysis of DHS data.”* The authors have submitted a detailed point-by-point response and made substantial revisions to the manuscript. Below is a summary of my evaluation based on the comments I previously raised:

The revised manuscript demonstrates strong improvements in clarity, methodological rigor, and contextual relevance. The title and abstract are well-structured; country selection and survey years are justified. Definitions, variable coding, and data access are clearly explained. The multilevel modeling approach is sound, with appropriate weighting, model comparison, and interpretation of random effects. Tables and formatting are improved. Key predictors of SBA ANC visits, education, wealth, media exposure, and residence are well contextualized. Ethical procedures are documented, and policy recommendations are relevant and actionable.

**However, the following points require further revision. Please consider reducing redundancy by using synonyms or restructuring sentences to enhance clarity and maintain a professional academic style.**

**Comment 1:** The phrase "skilled birth attendance" appears excessively (7 times in the abstract, 62 times overall), which may affect readability. Please try to address all redundant words like skilled birth attendance, extremely high maternal mortality

**Comment 2:** The phrase “extremely high maternal mortality” is overused (20 times); consider varying it for clarity. Also, “SBA” is underused despite being defined. After defining the acronym, consider using "SBA" more consistently to simplify the text and avoid repetition of the full phrase. Please try to address all redundant words

**Comment 3:** The term “reproductive-age women” appears 3 times in the abstract and 23 times in the full manuscript. Consider reducing repetition by using alternatives such as “women of childbearing age,” “target population,” or “study participants” where appropriate to enhance readability and stylistic variation

**Overall Assessment:**

The authors have satisfactorily addressed all the previous comments. The manuscript is now substantially improved in terms of clarity, methodological rigor, and contextual relevance. I recommend proceeding with editorial consideration.
