# Supplementary material for: Skilled birth attendance and its associated factors in Chad and Nigeria: A multilevel analysis of DHS data
Source: PLOS Glob Public Health. 2025 Dec 10;5(12):e0005290. doi: 10.1371/journal.pgph.0005290 (PMC12694868; doi:10.1371/journal.pgph.0005290)
Supplement: Attachment — Submitted filename: Point by Point response to reviwers.docx [file pgph.0005290.s006.docx]

**Authors’ Point-by-point response to editor and reviewer comments**

We are very grateful to both the editor and reviewers for your comments and suggestions. Appreciating your effort and valuable comments, we have provided corrections for the raised concerns and questions. Kindly find our reflections here. We hope you will consider the revised manuscript acceptable for publication in PLOSE Global Public Health Journal.

|  | **Reviewers’ comments** | **Authors’ responses** |
| --- | --- | --- |
| 1 | I have reviewed the revised manuscript titled “Skilled birth attendance and its associated factors in Chad and Nigeria: A multilevel analysis of DHS data.” The authors have submitted a detailed point-by-point response and made substantial revisions to the manuscript. Below is a summary of my evaluation based on the comments I previously raised: The revised manuscript demonstrates strong improvements in clarity, methodological rigor, and contextual relevance. The title and abstract are well-structured; country selection and survey years are justified. Definitions, variable coding, and data access are clearly explained. The multilevel modeling approach is sound, with appropriate weighting, model comparison, and interpretation of random effects. Tables and formatting are improved. Key predictors of SBA ANC visits, education, wealth, media exposure, and residence are well contextualized. Ethical procedures are documented,  and policy recommendations are relevant and actionable. However, the following points require further revision. Please consider reducing redundancy by using synonyms or restructuring sentences to enhance clarity and maintain a professional academic style. | We thank the reviewer for the positive evaluation of our revised manuscript. We appreciate the remaining comment regarding redundancy and clarity. |
| 2 | Comment 1: The phrase "skilled birth attendance" appears excessively (7 times in the abstract, 62 times overall), which may affect readability. Please try to address all redundant words like skilled birth attendance, extremely high maternal mortality | Dear reviewer, thank you for pointing it out. We have carefully reviewed the abstract and full manuscript and reduced redundancy by replacing repeated terms and reduced redundancy by replacing repeated terms with appropriate synonyms. These revisions have improved the manuscript’s readability and flow. Kindly see the whole revised manuscript on line 18, 19, 26, 28, 34, 36,38, 71,76, 88, 91,15… etc. |
| 3 | Comment 2: The phrase “extremely high maternal mortality” is overused (20 times); consider varying it for clarity. Also, “SBA” is underused despite being defined. After defining the acronym, consider using "SBA" more consistently to simplify the text and avoid repetition of the full phrase. Please try to address all redundant words | Thank you for this helpful observation. We have revised the manuscript to reduce overuse of the phrase “extremely high maternal mortality.” Additionally, after defining the term, we now use “SBA” consistently throughout the text to minimize repetition of the full phrase. These adjustments have improved clarity, conciseness, and overall readability. Kindly see the whole revised manuscript. |
| 4 | Comment 3: The term “reproductive-age women” appears 3 times in the abstract and 23 times in the full manuscript. Consider reducing repetition by using alternatives such as “women of childbearing age,” “target population,” or “study participants” where appropriate to enhance readability and stylistic variation | Thank you for noting this repetition. We have revised the manuscript by replacing repeated uses of “reproductive-age women” with suitable alternatives such as “women of childbearing age,” “study participants,” or “the target population,” and reduced the term reproductive -age women from 23 to 8 depending on context. Kindly see the revised manuscript on line 23, 88, 92, 191, 212, 215, 222, 228, 270, and 272. |
